# Focal Myocarditis after Mild COVID-19 Infection in Athletes

**DOI:** 10.3390/diagnostics11081519

**Published:** 2021-08-23

**Authors:** Ivana P. Nedeljkovic, Vojislav Giga, Marina Ostojic, Ana Djordjevic-Dikic, Tamara Stojmenovic, Ivan Nikolic, Nenad Dikic, Olga Nedeljkovic-Arsenovic, Ruzica Maksimovic, Milan Dobric, Nebojsa Mujovic, Branko Beleslin

**Affiliations:** 1 School of Medicine, University of Belgrade, 11000 Belgrade, Serbia; voja2011@yahoo.com (V.G.); skali.ana7@gmail.com (A.D.-D.); olganedeljkovic@gmail.com (O.N.-A.); dr.ruzica.maksiovic@gmail.com (R.M.); iatros007@gmail.com (M.D.); nmujovic@gmail.com (N.M.); branko.beleslin@gmail.com (B.B.); 2 Cardiology Department, University Clinical Center of Serbia, 11000 Belgrade, Serbia; drmarinaostojic@gmail.com; 3 Private Practice for Sports Medicine “Vita Maxima”, 11030 Belgrade, Serbia; antictamara@hotmail.com (T.S.); nikolic.ivan6@gmail.com (I.N.); nenad.dikic@gmail.com (N.D.); 4 Faculty of Physical Culture and Sports Management, Singidunum University, 11000 Belgrade, Serbia; 5 Radiology and MRI Department, University Clinical Center of Serbia, 11000 Belgrade, Serbia

**Keywords:** COVID-19 infection, athlete, return to play, myocarditis, cardiac magnetic resonance

## Abstract

COVID-19 infection in athletes usually has a milder course, but in the case of complications, myocarditis and even sudden cardiac death may occur. We examined an athlete who felt symptoms upon returning to training after asymptomatic COVID-19 infection. Physical, laboratory, and echocardiography findings were normal. The cardiopulmonary exercise test was interrupted at submaximal effort due to severe dyspnea in the presence of reduced functional capacity in comparison to previous tests. Cardiac magnetic resonance (CMR) detected the focal myocarditis. After three months of recovery, CMR still revealed the presence of focal myocarditis and the persistence of decreased functional capacity. This case raises the question of screening athletes even after asymptomatic forms of COVID-19 infection.

## 1. Introduction

Competitive sports, including training and matches, create favorable conditions for the spread of SARS-CoV-2 virus, which causes COVID-19 infection [1]. Recent recommendations for the evaluation of athletes who test positive for COVID-19 prior to return to play (RTP) were based on the presence of symptoms, electrocardiography (ECG), cardiac biomarkers, and echocardiography or cardiac magnetic resonance (CMR) imaging for advanced assessment [2,3]. Although COVID-19 infection in athletes usually has a milder course, in the case of complications, myocarditis and even sudden cardiac death may occur, as with myocarditis of other causes [4]. However, for athletes after mild or asymptomatic COVID-19 infections, no pre-RTP testing was scheduled. This pointed to the need to consider screening methods, but also that the recommendations must be applied flexibly due to the insidious course of this new disease, as recent CMR studies demonstrated a variable frequency of cardiac involvement in patients who recovered from asymptomatic to mild COVID-19 [5,6,7].

This case may not be unique, but it shows all of the diagnostic steps necessary to reach a diagnosis in an athlete after asymptomatic COVID-19 infection.

## 2. Case Report

A 32-year-old athlete received a positive PCR test for COVID-19 during routine testing of the entire team. Laboratory analysis showed normal findings (Table 1). As he was asymptomatic, isolation of 15 days was recommended. He received a permit to return to training once the isolation of 15 days was over and after confirmation of another negative COVID-19 test result. At the control visit, laboratory findings were still normal (Table 1).

**Figure 1:**
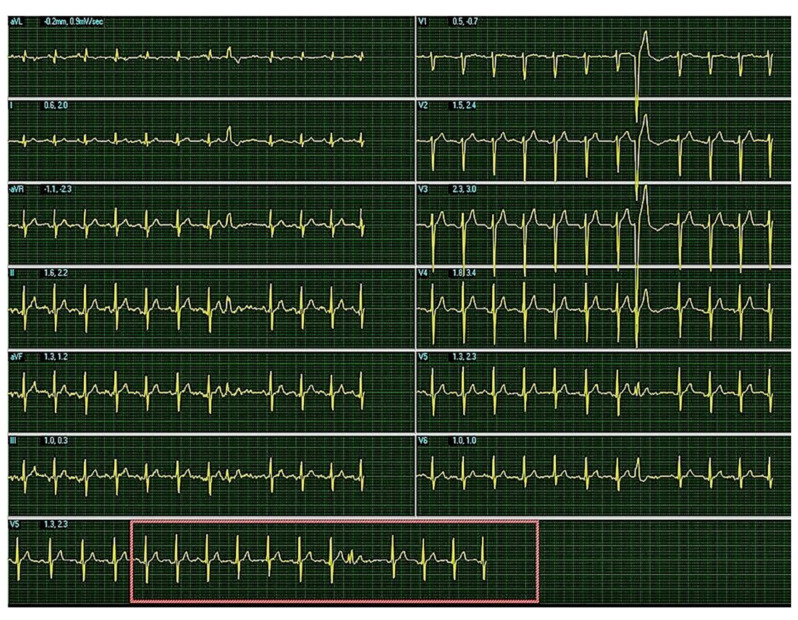
Resting ECG: sinus rhythm, heartrate = 95/min, AxQRS + 70, narrow QRS complex, R/S in leads D2, D3, aVF, r/S in leads V2, V3 with unifocal premature ventricular contraction with left bundle branch block morphology.

However, during the exercise, he suddenly became dyspneic and had a feeling of dizziness, which is why he was referred to a cardiologist. Physical examination showed normal findings and the resting ECG was recorded (Figure 1). The ECG showed a pathological finding compared to earlier routine controls, suggesting that the COVID-19 infection was not completely silent.

Although the acute phase of the infection itself was asymptomatic, ECG changes with an accelerated heartrate and the presence of PVC indicated an abnormality. We performed a treadmill cardiopulmonary exercise test (CPET) to assess functional capacity, hemodynamic response, and possible arrhythmias in exertion. We used the continuous sports ramp protocol. Expiratory gases were collected on a breath-by-breath basis and analyzed by metabolic cart (Schiller CS 200, Germany). The ventilatory anaerobic threshold (VAT) was determined by the “V-slope” analysis on oxygen consumption (VO_2_) vs. carbon dioxide production (VCO_2_). The values of the VO_2_ at VAT and at peak exercise (peak VO_2_) are expressed as mL O_2_/kg/min during the 30 s in which the examined event occurred, and were printed using rolling averages every 10 s. The ventilatory efficiency (VE/VCO_2_) slope was measured by excluding data points after the onset of maximal hyperventilation at the maximal effort. The respiratory exchange ratio (RER) ≥ 1.20 at the end of CPET was considered as the achievement of maximal effort. The test was interrupted at submaximal effort (RER = 1.06) due to the sudden onset of severe dyspnea, with achievement of only 76% of the predicted maxVO_2_ in the presence of normal ventilatory parameters. Compared with the previous test, which achieved maximum results, this result showed a decrease of exercise capacity after COVID-19 infection (Table 2).

**Figure 2:**
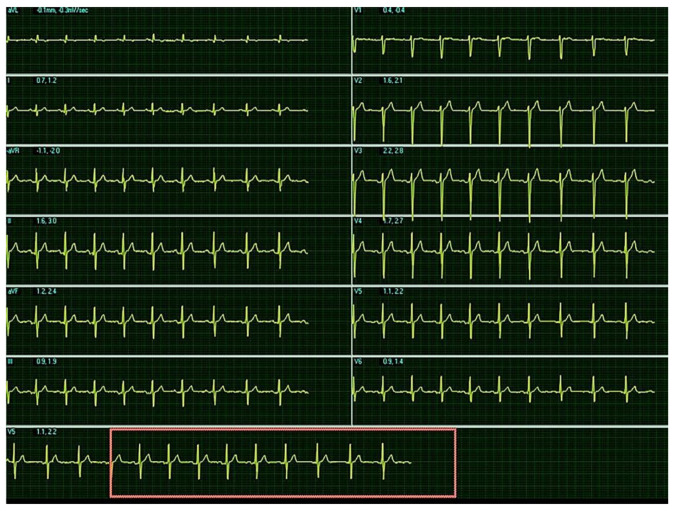
Baseline ECG before the cardiopulmonary test: sinus rhythm, heartrate = 80/min, AxQRS + 70, narrow QRS complex, R/S in leads D2, D3, aVF, r/S in leads V2, V3 without signs of hypertrophy.

**Figure 3:**
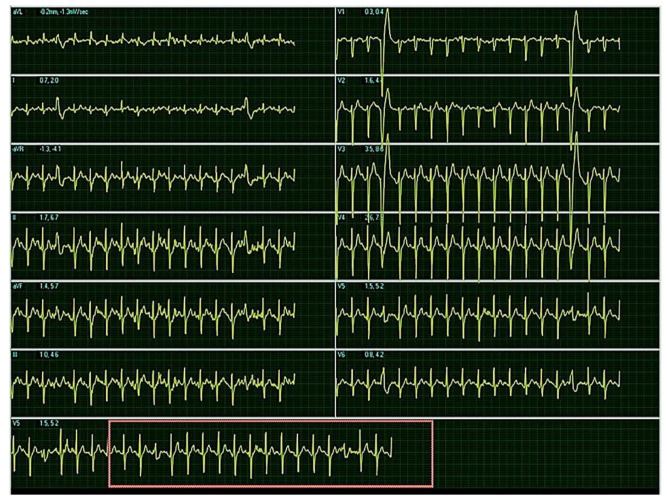
Electrocardiogram during cardiopulmonary test: sinus tachycardia, heartrate = 166/min, AxQRS + 70, narrow QRS complex, R/S in leads D2, D3, aVF, r/S in leads V2, V3 with unifocal premature ventricular complexes with left bundle branch block morphology. Ambulatory 24-h ECG monitoring recorded 1535 PVCs and several episodes of trigeminia.

The baseline ECG before CPET is presented on Figure 2. ECG during the test showed that more frequent exercise induced premature ventricular complexes (PVCs) (Figure 3).

**Figure 4:**
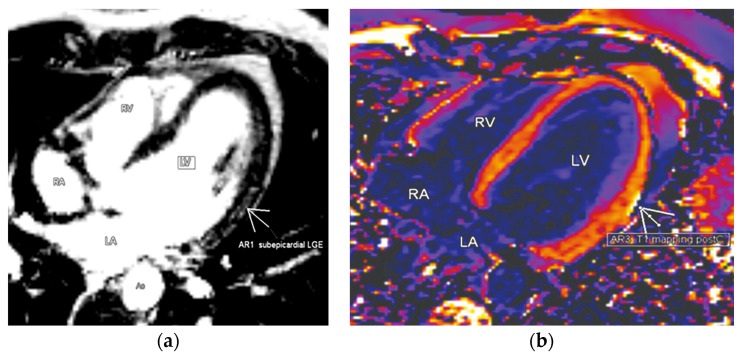
Cardiac magnetic resonance. (**a**) Four-chamber view ten minutes from the application of gadolinium with detection of the late gadolinium enhancement in the mid-lateral left ventricular wall. (**b**) Myocardial mapping (four-chamber view) showed elevated T1 signals of the mid-lateral left ventricular wall, with the enhancement of the adjacent pericardium also confirming the presence of pericarditis.

Cardiovascular magnetic resonance (CMR) was performed as the current non-invasive imaging gold standard for assessing cardiac anatomy, function, and more importantly, myocardial tissue characterization. We used standard planes with functional TrueFISP and morphological turbo spin-echo sequences. There were no signs of tissue edema in native images. Ten minutes after the application of contrast, we managed to detect late gadolinium enhancement (LGE) in the mid-lateral left ventricular (LV) wall (Figure 4a). Parametric myocardial mapping was also performed to add incremental diagnostic and prognostic value to conventional tissue characterization techniques in the evaluation of potential COVID-related myocardial diseases (Figure 4b).

Prescribed therapy included ibuprofen, bisoprolol, and coenzyme Q-10, with the cessation of training for the next three months.

**Figure 5:**
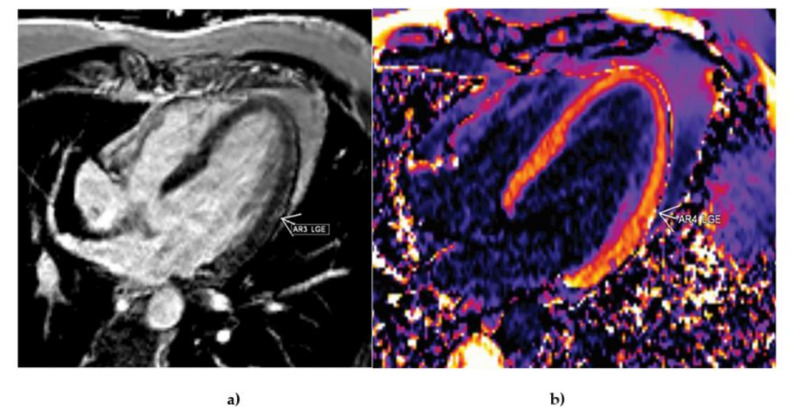
Control cardiac magnetic resonance three months after COVID-19 infection. (**a**) Persistence of late gadolinium enhancement in the basal and mid-lateral left ventricular wall. (**b**) Control CMR mapping detected increased values of postcontrast T2 subepicardial in the basal and medial part of the LV lateral wall in terms of myocarditis sequelae. The focal involvement of the pericardium along the lateral wall of the left ventricle can also be seen.

After three months of follow-up, the control CMR showed preserved left and right ventricular function (LVEF 61%, RVEF 65%) with a homogeneous myocardial structure in native sequences. There were persistent postcontrast pathological findings of the medial part of the LV lateral wall in terms of myocarditis sequelae (Figure 5a,b).

The control CPET reached the maximal effort (RER 1.2) without improvement in peak VO_2_ in the presence of normal ventilator parameters (Table 2). Further interruption of training has been recommended the next three months.

## 3. Discussion

Although hospitalization for acute COVID-19 infection is uncommon in athletes, it may result in myocarditis, even in the absence of symptoms with the rate of 2 to 3% [1,2,3]. However, in overt myocarditis, exercise may have a potentially pro-arrhythmic effect, which may lead to sudden cardiac death due to accelerated inflammation and cellular necrosis [3,4]. Routine cardiac testing after asymptomatic COVID-19 infection has not been recommended, but the occurrence of symptoms after RTP requires meticulous attention [1,2,3]. We present the case of an athlete who experienced symptoms after RTP while the previous COVID-19 infection itself had been silent. 

Reduced functional capacity during CPET in athletes indicates the need for evaluation with CMR, as it may detect the myocarditis in the form of LGE, but it does not itself represent a rationale for training restriction [8,9]. In our case with asymptomatic COVID-19 infection, reduced functional capacity provided additional clinical information to the CMR findings in terms of focal myopericarditis. This is in concordance with the cohort study of Daniels et al. of 1597 US competitive athletes with CMR screening after COVID-19 infection. They confirmed the role of CMR imaging for the detection of clinical and subclinical myocarditis in 2.3% of college athletes after COVID-19 infection [10].

Complex mechanisms of impaired functional capacity after COVID-19 cannot be attributed solely to focal myocarditis, but also to tissue abnormalities in other organs, such as the lungs and kidneys, as identified by CMR [7,8], which explains the reduced peak VO_2_ in our athlete in the presence of normal heart and lung function. Gao et al. also proposed CPET as a useful test to distinguish the reasons of effort intolerance after COVID-19, suggesting that it can be affected not only by ventilatory inefficiency, but also by cardiac involvement [11]. Furthermore, as in our case report, the pericardial enhancement even without effusion is strongly associated with COVID-19 pathological cardiac sequalae as described by [9]. Malek et al. suggested that pathological CMR in elite athletes should not be considered restrictive for RTP in the presence of normal ECGs and laboratories after a mild COVID-19 infection. Our athlete had reduced peak VO_2_ even after three months of recovery [12].

Thus, in the absence of symptoms and pathological cardiac biomarkers in athletes, a careful approach is required for appropriate clinical decision-making. This case emphasizes the importance of early cardiac assessment even after asymptomatic COVID-19 infection and the need for the integrated evaluation of functional parameters (CPET) and CMR results in light of clinical findings.

## Figures and Tables

**Table 1:** Laboratory findings during COVID-19 infection and after a 15-day isolation.

Parameter	Baseline	After Two Weeks	Normal Range
Leucocytes (10^9^ g/L)	6.4	8.1	3.4–9.7
Lymphocytes (10^9^ g/L)	2.4	3.3	1.2–3.4
Eosinophils (10^9^ g/L)	0.20	0.40	0.0–0.1
Red blood cells (10^9^ g/L)	5.56	5.23	3.86–5.08
Hemoglobine (g/L)	156	145	119–157
Platelets (10^9^ g/L)	220	165	158–424
Fe (mcmol/L)	15.8	15.4	7.0–28.0
Ferritin (mcg/L)	16.3	17	4.6–204.0
Creatinine (mcmol/L)	71	75	45–84
C-reactive protein (mg/L)	4	3.2	0.00–10.0
BNP ^1^ (pg/mL)	8	7	0–89
hs Troponin ^2^ T(µg/L)	7	8	<10
Interleukin 6 (pg/mL)	5	4	0–7
D-dimer (mg/L FEU)	0.28	0.36	<0.50
K ^3^ (mmol/L)	4.5	4.2	3.5–5.1
Na ^4^ (mmol/L)	140	135	
Lactate dehydrogenase (U/L)	376	350	220–460
AST ^5^ (U/L)	24	19	0–37
ALT ^6^ (U/L)	18	14	0–41

^1^ BNP—brain natriuretic peptide; ^2^ hs Troponin T—high sensitivity troponin T; ^3^ K—serum potassium; ^4^ Na—serum sodium; ^5^ AST—aspartate aminotransferase; ^6^ ALT—alanine aminotransferase.

**Table 2:** Parameters of cardiopulmonary exercise testing before COVID-19 infection in the athlete and after two weeks and three months of follow-up.

Variable	Before COVID-19 Infection	After Two Weeks of Follow-Up	After Three Months of Follow-Up
Test duration (s)	900	490	600
Resting heart rate (bpm)	50	80	85
Peak heart rate (bpm)	177	166	160
Resting systolic blood pressure (mmHg)	132	140	130
Peak systolic blood pressure (mmHg)	140	160	150
Resting diastolic blood pressure (mmHg)	75	75	70
Peak diastolic blood pressure (mmHg)	80	90	70
AT VO_2_ ^1^ (mL/kg/min)	26	23.1	22
Peak VO_2_(mL/kg/min)	45	32.2	31.4
% Predicted VO_2_	106	76	74
Peak VO_2_/HR ^2^ (mL/beat)	23	18	19
VE/VCO_2_ slope ^3^	24	27	20
Resting PetCO_2_ ^4^(mmHg)	31	30	33
Peak PetCO_2_ (mmHg)	49	48	45
METs ^5^	11.6	9.2	8.9
RER ^6^	1.2	1.06	1.2

^1^ AT VO_2_—oxygen consumption at the ventilatory anaerobic threshold; ^2^ VO_2_/HR—oxygen pulse; ^3^ VE/VCO_2_ slope—minute ventilation to carbon dioxide production relationship slope; ^4^ PetCO_2_—end-tidal carbon dioxide; ^5^ METs—metabolic equivalents; ^6^ RER—respiratory exchange ratio.

## Data Availability

Not applicable.
